# Incidence and Management of Pneumocephalus, an Uncommon Complication of Spinal Anesthesia: A Case Report

**DOI:** 10.1002/ccr3.71727

**Published:** 2026-04-16

**Authors:** Mohammad Saeidi, Sajjad Ahmadpour, Mohammad Amin Afshani, Fatemeh Sadat Razavinia, Reza Aminnejad

**Affiliations:** ^1^ Clinical Research Development Center Shahid Beheshti Hospital Qom Iran; ^2^ Patient Safety Research Center, Clinical Research Institute Urmia University of Medical Science Urmia Iran; ^3^ USREN Office, Functional Nerosurgery Research Center, Shahid Beheshti University of Medical Sciences Tehran Iran; ^4^ Clinical Research Development Center, Shahid Beheshti Hospital Qom Iran

**Keywords:** CT scan, headach, peripheral nerve blocking, pneumocephalus, spinal anesthesia

## Abstract

The presence of air in the epidural, subdural, or subcranial space within the brain parenchyma or ventricular cavities is a relatively uncommon side effect of spinal anesthesia. In this report, we aimed to describe a 37‐year‐old male patient who presented with continuous headaches, nausea, and vomiting during spinal anesthesia for joint replacement. A 37‐year‐old male patient was admitted for elective total hip arthroplasty. The surgery was performed under spinal anesthesia. Within 24 h after surgery, the patient presented with a history of continuous headaches, nausea, and vomiting. A CT scan of the brain was performed, and the findings revealed pneumocephalus, as the necessary measures were taken to control pneumocephalus. Finally, the patient was discharged from the hospital in good general condition. Pneumocephalus must be considered one of the possible causes of spinal anesthesia, which requires careful evaluation.

## Key Clinical Message

Pneumocephalus, though uncommon, is a critical differential diagnosis for severe headache following neuraxial anesthesia. Its presentation can mimic postdural puncture headache. A high index of suspicion is warranted, and early neuroimaging is crucial for confirmation, enabling timely conservative management and preventing potential neurological sequelae.

**Abbreviations**
CT: Computed tomography
LOR: Loss‐of‐resistance

## 1 Introduction

Pneumocephalus (also known as pneumatocele or intracranial aerocele) refers to the presence of air in the epidural, subdural, or subcranial space within the brain parenchyma or ventricular cavities, which was described by Lecat in 1741 [1]. The term “pneumocephalus” independently was described by Luckett in 1913 and Wolff in 1914 [2, 3]. To date, various etiologies have been reported for pneumocephalus, the most common of which are traumatic cases, such as fractures involving the skull base with a breach of the dura, penetrating head injuries with a dural laceration, high‐pressure trauma to the conjunctiva, and subarachnoid–pleural fistula. The prevalence of each is variable according to the etiology, and it is estimated to range from 1% to 82%. Infectious diseases and neoplastic conditions are the other etiologies of pneumocephalus [4, 5]. The entrance of air through the meninges following accidental injection, inadvertent dural puncture, or the differential pressures between the cranial cavity and atmosphere constitute one of the etiologies of pneumocephalus. Pneumocephalus can be caused by the diffusion of nitrous oxide during anesthesia into the cranial cavity faster than the diffusion of nitrogen or air [6].

One of the main challenges of medical science is the clinical diagnosis of pneumocephalus, which is rare in rare cases, following the history of hydroaerique bruit, which involves hearing splashing sounds in the head that can be heard. In addition, one of its common clinical symptoms is a sudden and very severe headache, which in some cases cannot be improved or controlled by changing the state [7]. CT is the gold standard diagnostic modality for pneumocephalus and is effective in identifying low air in the skull, even as low as 0.55 mL, whereas the diagnosis of pneumocephalus by skull radiography requires at least 2 mL of air [8]. The purpose of this study was to introduce the case of a 37‐year‐old man who experienced a sudden severe headache after spinal anesthesia, and after investigations, he was diagnosed with pneumocephalus, which is one of the relatively uncommon side effects of spinal anesthesia.

## 2 Case History/Examination

A 37‐year‐old male patient was referred to Shahid Beheshti Hospital in Qom Province for hip joint replacement. The patient's vital signs at admission were as follows: BP: 110/70 mmHg, HR: 60/min, RR: 14/min; SpO2 (Room Air): 96%, and the patient's history of drug use and allergy was negative. The patient underwent the procedure under spinal anesthesia. The patient was positioned in the left lateral decubitus position. Under aseptic conditions, loss‐of‐resistance (LOR) to air was used to identify the epidural space at the L3‐L4 interspace with an 18‐gauge Tuohy needle. During this procedure, an inadvertent dural puncture was noted with return of clear cerebrospinal fluid. The Tuohy needle was withdrawn. Subsequently, a spinal anesthetic was successfully performed at the L4‐L5 interspace using a 25‐gauge Quincke needle with the injection of 12.5 mg of 0.5% hyperbaric bupivacaine. The surgery then proceeded uneventfully. The history of the patient's surgery was as follows: the duration of the surgery was 1.5 h, and during the surgery, he experienced 400 cc of bleeding and 400 cc of urine output, and he received 2.5 L of normal saline and Ringer's serum. After recovering from stable vital signs, the patient recovered when his clinical conditions were controlled. During hospitalization within 24 h after surgery, the patient presented with a history of continuous headaches and nausea and vomiting; thus, both anesthesia and neurology consultations were requested.

## 3 Differential Diagnosis, Investigations and Treatment

**FIGURE 1:**
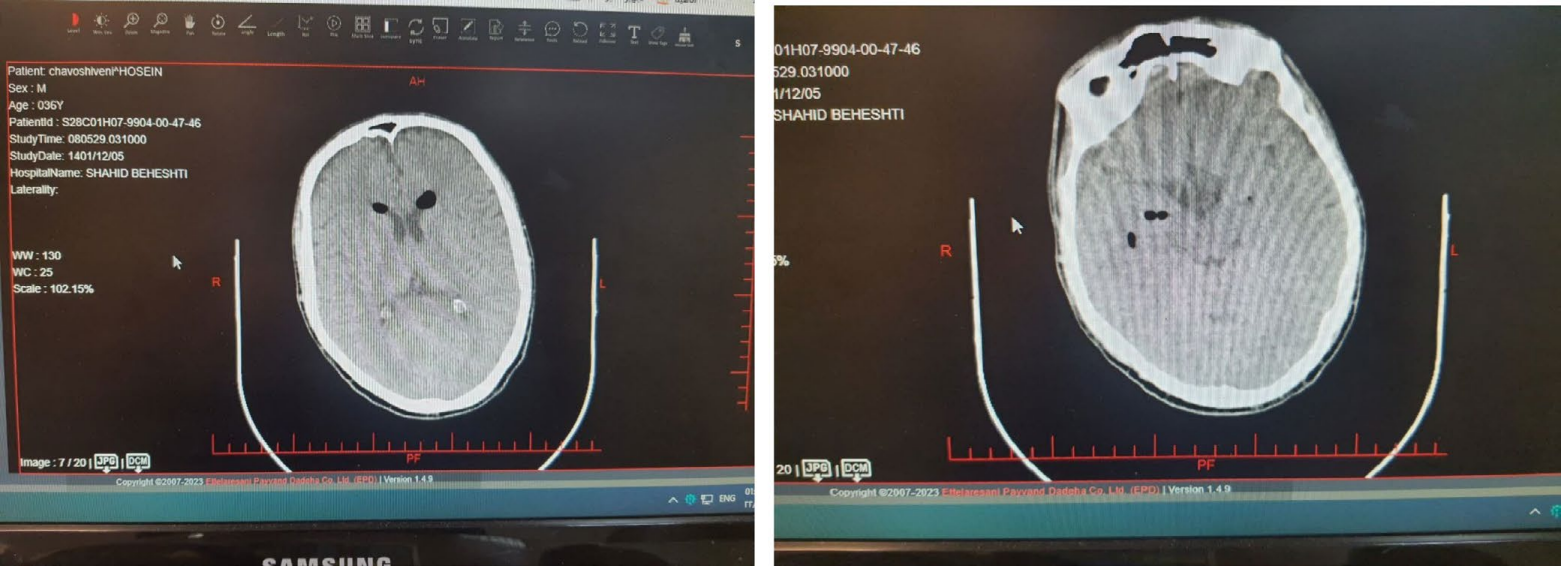
Axial noncontrast computed tomography (CT) images of the brain in a 37‐year‐old male patient who developed pneumocephalus following epidural anesthesia for hip joint replacement. Multiple small intracranial air foci are visible within the frontal region and subarachnoid space, which is consistent with pneumocephalus. No evidence of intracranial hemorrhage or midline shift were observed.

For precise diagnosis, a CT scan of the brain was performed without contrast, and the results revealed pneumocephalus in the CT scan (Figure 1). In this way, the necessary measures were taken to control pneumocephalus, as the patient was discharged from the hospital in good general condition.

## 4 Conclusion and Results (Outcome and Follow‐Up)

In this study, we described a patient who experienced a severe headache during hospitalization. A precise evaluation confirmed pneumocephalus, the patient underwent symptomatic treatment, and the headache improved. In conclusion, this report underscores that pneumocephalus is not merely a complication of dural puncture, but more specifically, a potential consequence of the LOR to air technique when such a puncture occurs, necessitating heightened clinical vigilance in this scenario.

## 5 Discussion

Spinal anesthesia is an effective anesthesia technique that is created by perforating the skin of the epidural space (also known as the extradural space or peridural space), subcutaneous tissue, supraspinous and intraspinous ligaments, flavum ligaments and, finally, the epidural space; it is used as a complementary method to general anesthesia or the primary anesthetic approach in orthopedic surgery of the lower limb [9]. In addition, in some patients with peripheral nerve blocking, this procedure significantly reduces postoperative pain. Orthopedic surgeries such as total hip arthroplasty or THA, total knee arthroplasty, foot/ankle surgery, and major knee surgery are the most important indications for spinal anesthesia [10]. Notably, its absolute contraindications include patient dissatisfaction, local infection at the spinal anesthesia induction site, and severe coagulation disorders. Other contraindications include sepsis, increased intracranial pressure, anticoagulant drugs, bleeding disorders, fever, and aortic stenosis [11].

Spinal anesthesia is more suitable for older patients with underlying heart diseases who cannot tolerate the sudden blockade of the sympathetic system and blood pressure reduction caused by spinal anesthesia because the injection of anesthetic gradually uses an epidural catheter, and the possibility of major rapid changes in blood pressure is significantly reduced. Additionally, the epidural catheter can be maintained until the end of surgery and can be used for other medications, such as controlling the patient's pain or inflammation; its advantage is greater than that of other anesthesia procedures [12, 13]. Notably, spinal anesthesia, like other medical procedures, is associated with various complications. The prevalence of serious complications of spinal anesthesia is very rare, estimated to be less than 1%, but nevertheless, it may be serious and life‐threatening. These complications include epidural hematoma, epidural abscess, vascular damage, infection and instability of the cardiovascular system [14]. Although some data support the association between pneumocephalus and injection in the epidural space, findings do not reveal pneumocephalus complications in spinal anesthesia, and very limited information exists in this field. Koo et al. reported a case of a 60‐year‐old woman who presented with a thunderclap headache and febrile sensation 3 h after lumbar interlaminar epidural steroid injections [15]. A brain CT scan revealed multiple small foci of air within the subarachnoid space and ventricles, confirming the diagnosis of pneumocephalus. This case, similar to our own, underscores that pneumocephalus is a potential complication of procedures involving inadvertent dural puncture and must be included in the differential diagnosis for a post‐procedural headache.

While previous reports have established a link between neuraxial procedures and pneumocephalus [7, 15], the present case provides a clear and instructive delineation of the causative mechanism. The rapid onset of symptoms in our patient (within 24 h) is a classic presentation when a significant volume of air is introduced. The critical, and often underemphasized, risk factor demonstrated here is the use of air for LOR. We hypothesize that during the initial epidural attempt, a substantial volume of air was injected into the epidural space. The subsequent inadvertent dural puncture, a known complication, then created a direct communication, allowing the entrapped epidural air to rapidly enter the subarachnoid space. This mechanism is supported by literature suggesting that the LOR‐to‐air technique may carry a higher risk for this complication compared to saline [16]. The patient's positioning and the buoyancy of the air then facilitated its cranial ascent, leading to the symptomatic pneumocephalus. Therefore, our case underscores that the choice of technique for identifying the epidural space is not trivial and can be the primary determinant in the pathogenesis of pneumocephalus when a dural breach occurs.

## Author Contributions

**Mohammad Saeidi:** writing – original draft. **Sajjad Ahmadpour:** writing – review and editing. **Mohammad Amin Afshani:** data curation. **Fatemeh Sadat Razavinia:** visualization. **Reza Aminnejad:** supervision.

## Funding

No funding was provided for this manuscript.

## Ethics Statement

This study was approved by the Ethical Committee of Qom University of Medical Sciences (Ethical Code: IR.MUQ.REC.1403.209). The procedures used in this study adhered to the tenets of the Declaration of Helsinki.

## Consent

The patients signed informed consent regarding the publication of their data and photographs.

## Conflicts of Interest

The authors declare no conflicts of interest.

## Data Availability

All data and materials are available from the corresponding author.

## References

[1] M. J. Álvarez‐Holzapfel , J. Aibar Durán , S. Brió Sanagustin , and C. De Quintana‐Schmidt , “Diffuse Pneumocephalus After Lumbar Stab Wound,” Anales de Pediatria 90, no. 1 (2019): 63–64. 29903638 10.1016/j.anpedi.2018.04.012

[2] C. M. Schirmer , C. B. Heilman , and A. Bhardwaj , “Pneumocephalus: Case Illustrations and Review,” Neurocritical Care 13, no. 1 (2010): 152–158. 20405340 10.1007/s12028-010-9363-0

[3] H. Yates , M. Hamill , C. O. Borel , and T. J. Toung , “Incidence and Perioperative Management of Tension Pneumocephalus Following Craniofacial Resection,” Journal of Neurosurgical Anesthesiology 6, no. 1 (1994): 15–20. 8298259 10.1097/00008506-199401000-00002

[4] M. L. Biller , M. Böhm , C. Kolb , J. Bucur , M. Müller , and T. Kohnen , “Pneumocephalus After High‐Pressure Trauma to the Conjunctiva,” Die Ophthalmologie 120, no. 6 (2023): 660–662. 35925342 10.1007/s00347-022-01671-x

[5] E. Burkhardt , A. Savardekar , and A. Sin , “Traumatic Subarachnoid‐Pleural Fistula With Pneumocephalus,” World Neurosurgery 167 (2022): 229.e3. 35917920 10.1016/j.wneu.2022.07.080

[6] A. R. Clement , D. Palaniappan , and R. K. Panigrahi , “Tension Pneumocephalus,” Anesthesiology 127, no. 4 (2017): 710. 28537932 10.1097/ALN.0000000000001703

[7] S. Reddi , V. Honchar , and M. S. Robbins , “Pneumocephalus Associated With Epidural and Spinal Anesthesia for Labor,” Neurology: Clinical Practice 5, no. 5 (2015): 376–382. 29443169 10.1212/CPJ.0000000000000178 PMC5762022

[8] E. Karavelioglu , O. Eser , and A. Haktanir , “Pneumocephalus and Pneumorrhachis After Spinal Surgery: Case Report and Review of the Literature,” Neurologia Medico‐Chirurgica 54, no. 5 (2014): 405–407. 24305016 10.2176/nmc.cr2013-0118 PMC4533435

[9] A. Rezayi Soufiani , M. Joulani , M. S. Jolani , and M. Parish , “Accessing the Efficacy and Peri‐Operative Adverse Effects of Three Different Hyperbaric Bupivacaine 0.5% Dosages for Spinal Anesthesia Induction in Lower Limb Orthopedic Surgeries: A Randomized Clinical Trial,” BMC Anesthesiology 24, no. 1 (2024): 285. 39134965 10.1186/s12871-024-02673-9 PMC11318273

[10] I. Kamel , M. F. Ahmed , and A. Sethi , “Regional Anesthesia for Orthopedic Procedures: What Orthopedic Surgeons Need to Know,” World Journal of Orthopedics 13, no. 1 (2022): 11–35. 35096534 10.5312/wjo.v13.i1.11 PMC8771411

[11] E. M. Helander , A. J. Kaye , M. R. Eng , et al., “Regional Nerve Blocks—Best Practice Strategies for Reduction in Complications and Comprehensive Review,” Current Pain and Headache Reports 23 (2019): 1–9. 31123919 10.1007/s11916-019-0782-0

[12] L. E. Imbelloni , M. A. Gouveia , and J. A. Cordeiro , “Continuous Spinal Anesthesia Versus Combined Spinal Epidural Block for Major Orthopedic Surgery: Prospective Randomized Study,” São Paulo Medical Journal 127 (2009): 7–11. 19466288 10.1590/S1516-31802009000100003 PMC10969317

[13] V. Tummala , L. N. Rao , M. K. Vallury , and A. Sanapala , “A Comparative Study‐Efficacy and Safety of Combined Spinal Epidural Anesthesia Versus Spinal Anesthesia in High‐Risk Geriatric Patients for Surgeries Around the Hip Joint,” Anesthesia, Essays and Researches 9, no. 2 (2015): 185–188. 26417125 10.4103/0259-1162.153764 PMC4563971

[14] D. S. D. Rusmilia and D. Indrayani , “Counter Pressure Untuk Mengurangi Rasa Nyeri Persalinan (Evidence Based Case Report): Indonesia,” Jurnal Kesehatan Siliwangi 3, no. 2 (2022): 196–204.

[15] J. Koo and K.‐T. Cho , “Pneumocephalus and Chemical Meningitis After Inadvertent Dural Puncture During Lumbar Epidural Injection,” Korean Journal of Neurotrauma 16, no. 1 (2020): 67–72. 32395453 10.13004/kjnt.2020.16.e8 PMC7192798

[16] S. K. Jung and K. H. Park , “Pneumocephalus After Epidural Steroid Injection: A Case Report,” Journal of the Korean Pain Society 14, no. 2 (2001): 276–279.

